# Hypothesis exploration with visualization of variance

**DOI:** 10.1186/1756-0381-7-11

**Published:** 2014-07-02

**Authors:** Douglass Stott Parker, Eliza Congdon, Robert M Bilder

**Affiliations:** 1 Computer Science Department, University of California, Los Angeles, CA, USA; 2 Department of Psychiatry and Biobehavioral Sciences, University of California, Los Angeles, CA, USA; 3 Semel Institute for Neuroscience and Human Behavior, University of California, Los Angeles, CA, USA

**Keywords:** Methodologies, Hypothesis generation and refinement, Visualization, EDA, ANOVA, Covariance structure

## Abstract

**Background:**

The Consortium for Neuropsychiatric Phenomics (CNP) at UCLA was an investigation into the biological bases of traits such as memory and response inhibition phenotypes—to explore whether they are linked to syndromes including ADHD, Bipolar disorder, and Schizophrenia. An aim of the consortium was in moving from traditional categorical approaches for psychiatric syndromes towards more quantitative approaches based on large-scale analysis of the space of human variation. It represented an application of phenomics—wide-scale, systematic study of phenotypes—to neuropsychiatry research.

**Results:**

This paper reports on a system for exploration of hypotheses in data obtained from the LA2K, LA3C, and LA5C studies in CNP. ViVA is a system for exploratory data analysis using novel mathematical models and methods for *visualization of variance*. An example of these methods is called *VISOVA*, a combination of visualization and analysis of variance, with the flavor of exploration associated with ANOVA in biomedical hypothesis generation. It permits visual identification of phenotype profiles—patterns of values across phenotypes—that characterize groups. Visualization enables screening and refinement of hypotheses about variance structure of sets of phenotypes.

**Conclusions:**

The ViVA system was designed for exploration of neuropsychiatric hypotheses by interdisciplinary teams. Automated visualization in ViVA supports ‘natural selection’ on a pool of hypotheses, and permits deeper understanding of the statistical architecture of the data. Large-scale perspective of this kind could lead to better neuropsychiatric diagnostics.

## Background

### Motivation: better neuropsychiatric diagnostics

Diagnosis in neuropsychiatry rests on an elaborate taxonomy of syndromes and explicit decision trees for classification. For example, these decision trees have been codified in DSM-IV [1] and its very recent revision DSM-V [2]. Dissatisfaction with the current situation has been evident throughout the development of DSM-V, and particularly its inclusion of dimensional classification (quantitative approaches to assessment and diagnosis) [3] as a step beyond DSM-IV ‘chinese menu’ diagnosis and categories that do not always fit, toward quantitative assessments of severity and treatment response that are grounded in data. However the benefits of dimensional approaches remain controversial. The stakes involved in this evolution are breathtaking, as the DSM is a cornerstone of the mental health system.

Many research efforts are now seeking better models for existing categories such as ADHD, Bipolar Disorder, and Schizophrenia. These rubrics are often said to be inadequate for classification because they rest on inaccurate descriptions. The NIMH Strategic Plan seeks improvements on existing diagnostic categories for mental disorders, both because the categories lack validity and because they limit incorporation of new scientific results. A criticism often leveled against the DSM is that it is not aligned with any scientific model of neuropsychiatric disorders [4]. A related criticism is that different diagnoses overlap significantly, in some cases using different terminology for the same concept.

Large databases can support both statistical evaluation of these criticisms and development of better diagnostics. For example, a recent investigation of differences in disorder incidence rates by gender [5] focused on patterns of disorder comorbidity based on the very large (*n* = 43,093) National Epidemiologic Survey on Alcohol and Related Conditions (NESARC). As discussed later, the results offered an overall statistical outline or architecture for disorders, clarifying how they impact men and women differently: women have a higher incidence of internalizing (mood and anxiety) disorders, while men have a higher incidence of externalizing (antisocial and substance use) disorders [6]. This link between gender on disorders has become formalized in the Internalizing-Externalizing ‘meta-structure’ of DSM-V [2], and the database analysis results in [5] suggest an important way it can be refined.

A related trend is the development of increasingly sophisticated models based on data [7]. General linear models (GLMs [8]) or structural equation models (SEMs [9], often referred to as CSA—covariance structure analysis) are becoming common, permitting characterization of variance structure with a set of functional equations. An emphasis on dimensional models has been developed within the NIMH Research Domains Criteria (RDoC) project [10,11], particlarly for diagnosis, permitting continuous variables of function, ranging from behavior down to neurobiology. The variance structure models and phenotype profiles emphasized in this paper are consistent with this trend. With large databases, these models have the potential for significant advances in neuropsychiatric diagnosis.

### The CNP database

The LA2K study [12,13] was conducted at UCLA during 2008–2012, designed within CNP as a large-scale analysis for about 2000 volunteers from the Los Angeles metropolitan region. Behind its development was the hypothesis that there may be sufficient variance in healthy people along dimensions shared with people with psychopathology, that we might find common mechanisms with genetic links. For example, the genetic bases for variability in working memory in healthy people may also be the basis for working memory impairments commonly found in patients. LA2K focused on the evaluation of memory and response inhibition as central endophenotypes [14] with the potential to serve as basic dimensions for neuropsychiatry. Ultimately this approach also could permit development of statistical models characterizing neuropsychiatric syndromes, without relying on traditional taxonomies and their discrete categories.

LA2K was developed in part, then, as a demonstration of the use of phenomics as a framework for neuropsychiatry, and hopefully also a way to advance the pace of research [15]. Phenotypes—detectable or measurable characteristics of an organism—are outward manifestations of interaction of its genotype and environment. Phenomics is the systematic study of biological and behavioral phenotypes [13]. Because phenotypes are present in all scales of science, phenomics is interdisciplinary and intrinsically large-scale in scope. LA2K was a 6-year effort aimed at discovery of relationships that would go undetected with a smaller scope. Initially LA2K obtained data on approximately 1300 subjects (healthy control subjects, not suffering from any major syndrome). The related LA3C and LA5C studies subsequently included some patients diagnosed with ADHD, Bipolar Disorder, and Schizophrenia, increasing opportunities for discovery.

Results from these studies are stored together in the CNP database, a relational database with about 50 tables together comprising over 2500 numeric variables (columns). Each column representing a single (but not independent) phenotype or measurement, and each table reflects an experimental protocol. Table 1 shows names of cognitive tasks and personality rating scales represented in the tables.

**Table 1:** Tasks and Tests in the LA2K database

**CNP database domain/Test**	**Abbrev**
*Consent/Screening/Diagnosis/Clinical Rating Scales*	
Adult ADHD Interview (module from KSADS-PL)	AAI
Hopkins Symptom Checklist 25	HSCL25
Structured Clinical Interview for DSM-IV/Axis I/Patient version	SCID-I/P
*Personality/Temperament/Symptom Questionnaires*	
The Temperament and Character Inventory	TCI
The Chapman Scales – Physical Anhedonia	RPAS
The Chapman Scales – Social Anhedonia	RSAS
The Chapman Scales – Perceptual Aberrations	PAS
Eckblad and Chapman’s Hypomanic Personality Scale	HPS
Golden and Meehl’s 7-Item Schizoid Scale	G+M
Munich Chronotype Questionnaire	MCTQ
Akiskal’s Bipolar II Scale	BPII
Barratt Impulsivity Scale	BIS
Eysenck’s Impulsivity Venturesome and Empathy Inventory	IVE-R
MPQ (Control-Impulsivity items)	MPQ-CI
The Dickman Scale of Functional vs Dysfunctional Impulsivity	DSFDI
*Neurocognitive measures*	
Spatial and Verbal Memory and Manipulation Tasks	SMNM/VMNM
Spatial and Verbal Working Memory Capacity Tasks	SCAP/VCAP
Remember-Know Paradigm	RK
Scene Recognition Task	SR
California Verbal Learning Test	CVLT-II
WMS-III Spatial Span	WMS-SS
WMS-III Digit Span	WMS-DS
WMS-III Visual Reproduction (part I/II [immed/delayed recall])	WMS-VRI
WMS-III Letter Number Sequencing	WMS-LNS
Stop-Signal Task	SST
Conner’s CPT II	CPT-II
Reversal Learning	PRLT
Task Set Switching	TS
Stroop Test	SCWT
Attention Networks Task	ANT
Delay Discounting	DDT
Balloon Analog Risk Task	BART

A list of tasks and tests represented as tables in the CNP database. Altogether the database has about 50 tables and 2500 variables (columns), with complete records for about 1300 subjects in the LA2K, LA3C, and LA5C studies.

In general terms, the CNP database permits investigators to analyze the behavior of subjects in neurocognitive measures that reflect memory and response inhibition (recording reaction times and accuracy measures), as well as aspects of temperament, personality, and syndromal behavior. Each table in Table 1 has data for the relevant subjects.

### Phenomic analysis

The LA2K study was designed around a schema spanning 7 levels of neuroscience, from genome to syndrome, centering on memory and response inhibition phenotypes [13]. The levels were designed to facilitate development of new models for syndromes, with hypotheses based on these phenotypes.

**Figure 1:**
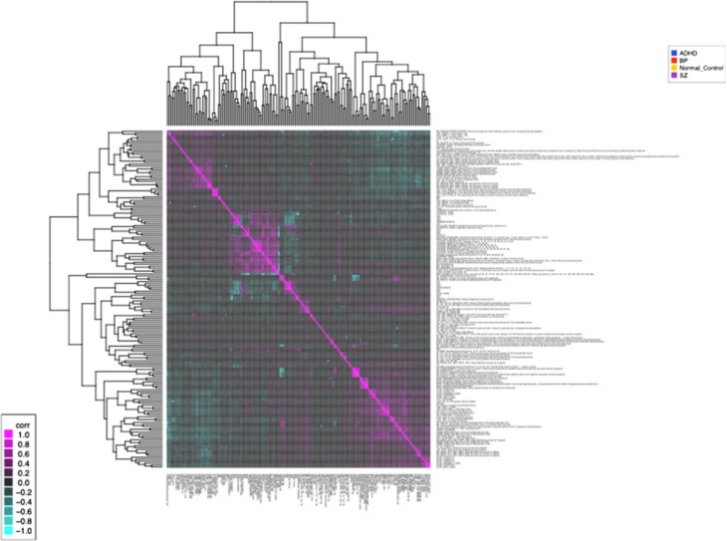
**The (clustered) correlation matrix for a set of more than 150 key variables that were carefully selected to represent a significant part of the variance in the data.** The dendrograms cluster variables by their correlation matrix similarity.

Traditional hypothesis testing can be difficult in phenomics. The diversity of variables and complexity of the systems they represent can challenge any experimental design. Analyzing variance in a set of phenotypes over different population groups requires perspective and an integration of knowledge that may not exist a priori. One consequence of integrative analysis is that hypothesis formulation can become more adaptive, with hypotheses evolving as perspective is gained [16]. Another is that visualization can become valuable for interpreting the breadth of phenomic information [17-19].As an illustration of the power of perspective, Figure 1 shows a correlation matrix for a set of over 150 key variables—a collection of phenotypes chosen by investigators as representing much of the variance. The matrix exhibits limited pockets of positive correlation, reflecting the careful selection of these variables so as to be independent. Generally the matrix shows low correlation outside these pockets—except some anti-correlation between variables at the start (reaction time variables) and variables at the end (total raw score variables), possibly reflecting speed-accuracy tradeoffs.

The dendrograms in Figure 1 show block structure, and suggest how the variables could be partitioned into clusters representing dimensions of significant variance. These clusters may correspond to *phenotype profiles*—patterns of values across sets of phenotypes that characterize important categories. A benefit of using a phenomics approach is that we may be able to identify useful profiles, and also be able to explain the variance in the population with models. For example, methods like PCA [20] might be used to summarize the variables in this matrix with about 20 dimensions, since the first 20 components explain about 50% of its variance.

**Figure 2:**
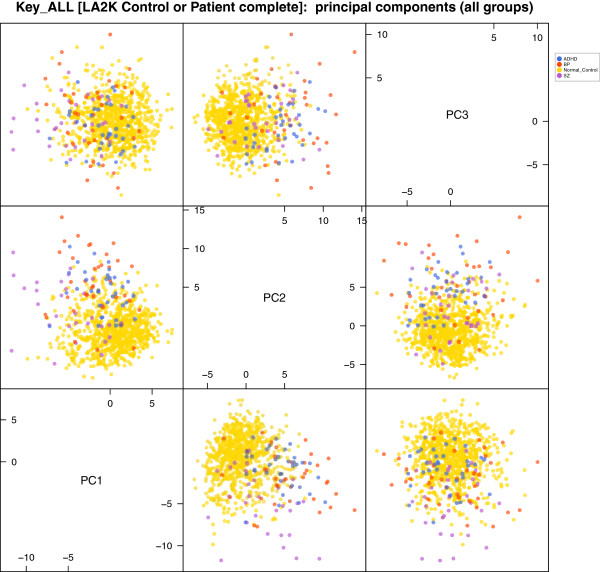
**The projection of the study data on the first 3 principal components **[20]** for the correlation matrix in Figure **1. Two views are offered—pairwise projections and a 3D view—showing the relative positions of healthy controls (light orange), ADHD patients (blue), Bipolar patients (red), and Schizophrenia patients (purple). Schizophrenia patients have extreme coefficient values on the first two principal component, and both BP and ADHD patients on the second and third. In the 3-dimensional perspective the separation of these four classes is more visible, with patients as outliers with high phenotypic variance. Very roughly, the three components respectively emphasize total raw scores in tasks, scores measuring psychological stress and mental health, and scores reflecting working memory performance.

However, decomposition ultimately requires conditioning on features of the population. Multi-level models permit conditioning on group or factor features such as gender or age, or syndromes like *ADHD*, *BP*, or *SZ*. This blend of categorical and quantitative modeling permits explanation of differences in variance for different population groups. An example using PCA is shown in Figure 2, illustrating how variance in the data can change if we condition on either the *ADHD*, *BP*, or *SZ* group. Multi-level variance structure is fundamental to analysis of variance (ANOVA) [8], and linear modeling that spans groups. Models like these can help map out the space of human variation.

### Variance structure models

*Variance structure* is a common term in data analysis. It typically refers to patterns of variation in statistical models related to distributions (variance of individual variables), covariance and correlation of variables, and more general equational relationships among variables. A *variance structure model* is a mathematical expression of these relationships.

Important classes of variance structure models include general linear models (GLMs [8]**) and structural equation models (SEMs **[9]). These permit characterization of variance structure in terms of a set of functional equations that are often linear in form. However they permit nonlinear interactions (nested models) and conditioning on non-numeric variables or factors that take discrete values (multi-level models).

Variance structure is often exposed by an incremental process of decomposition or factoring, yielding a hierarchy or graph of components that together form an overall model. For example, PCA is a linear algebraic model of covariance structure. From the standpoint of decomposition, this process also can differentiate clusters of similar variables from others, and extract hierarchical block structure.

An important aspect of variance structure is that it can often be visualized. For example, it is not a coincidence that the hierarchical model foundation for the popular *trellis graphics* visualization framework [21] corresponds to grids of graphics that highlight variation. This insight was the spark that led to the *ViVA (Visualization of VAriance)* system architecture described in this paper.

In ViVA, a variance structure model

M:Y∼X∣G:(S)

is a specification of a rough hypothesis with five items: 

● a subset *S* of the data (a population of subjects that is meaningful for analysis).

● a grouping *G*, specifying a set of class names (factor levels) defining several subpopulations.

● zero or more dependent variables *Y* from the CNP database. (If zero, all variables are independent).

● one or more independent variables *X* from the CNP database.

● a model of variance *M* (a mathematical model and/or visualization method for explaining variance).

In particular, the model

VISOVA:∼ReactionTime∣Age:(LA2K control)

relates reaction time to age groups that define population subsets—in this case restricted to control subjects. Multi-level models with this kind of conditioning are basic to ANOVA, explaining variance across the different values of a factor, such as the Female and Male values of *Gender*.

Although ViVA supports development of hypotheses about the study data, it deliberately has no facilities for traditional statistical hypothesis testing. This resolves a tension between discovery and hypothesis testing. A recent review [22]** contrasts ‘-ology’ strategies that typically test a priori hypotheses with ‘-omics’ strategies that adopt more agnostic, exploratory approaches, stressing that “... discovery-based approaches do not eschew hypotheses; rather, they seek to elevate hypothesis testing to a new level, by allowing high-throughput hypothesis generation and prioritization” **[22].

ViVA focuses on exploration of variance structure hypotheses in a large phenotype database. Exploration of hypotheses can involve analytical generation and prioritization, but a particular strength of ViVA has been as a source of ‘natural selection’ in a hypothesis pool. Grounding hypotheses in data—checking them as assertions about the CNP database—permits screening of weaker hypotheses and refinement of hypotheses that survive. With this kind of testing, the process of hypothesis generation [16] becomes one of evolution.

### Objectives of this paper

This paper describes ViVA’s support for exploring hypotheses. The system was designed for neuropsychiatric hypothesis exploration by interdisciplinary teams, and more specifically for grounding them in data (checking them as concrete assertions about LA2K). Automatically-constructed visualizations in ViVA permit exploration and deeper understanding of variance structure, and grounding improves the hypothesis pool. With a sequence of examples, we illustrate how this approach can contribute towards the development of better neuropsychiatric diagnostics.

The system is novel in several ways. First, ViVA directly links visualization with variance structure models, permitting interactive visual exploration of natural models in a large database. It includes *VISOVA*, for example, a combination of visualization and analysis of variance with the exploratory flavor that is associated with ANOVA in biomedical hypothesis generation. Viewing variance structure models as hypotheses, it provides automated support for integrative analysis of large phenotype databases. The examples given attempt to illustrate its potential in developing greater understanding of phenotypic variation.

## Methods

This project initially began as a server aimed at sharing of exploratory data analysis scripts for the CNP database. These evolved, until it eventually became clear that they emphasized visual analogues of ANOVA—including what we call *VISOVA*—holding some variables fixed, varying others, and permitting visualization of the resulting ‘response’.

### ViVA architecture

ViVA is actually three related systems, each providing a different mode of exploration: 

● *ViVA Atlas*—‘gallery’ of results for predefined hypotheses.

● *ViVA Viewer*—simple menu-based hypothesis exploration.

● *ViVA Explorer*—advanced development of hypotheses.

These three differ mainly in the power of the interface. The *Atlas* has a simple interface, allowing immediately access to wide sets of results, with large pre-computed reports that overview the data. The *Viewer* allows relation-level exploration, permitting rapid visual exploration of entire tables listed in Table 1. The *Explorer* has an advanced interface, permitting definition of population groups and analysis of specific variables. These three systems can be used in sequence, progressing from initial passive orientation to active exploration.

The architecture common to these three systems aims at making exploration as effortless as possible. This boils down to automation: automated data cleaning, implementation of group tables, creation of tables and fields wherever needed, development of web infrastructure, and introduction of features aimed at simplifying interaction and rising above the complexity of the database.

Experience with ViVA has highlighted benefits of this architecture for visualization of variance: 

● implementing visualization with a server has advantages for pooling effort (such as in data cleaning and visualization scripts), and for maintaining best practices and standards (such as verification of distributions).

● using universally-understood vocabularies of visualization and exploratory data analysis (EDA [23] and ANOVA) can help in trans-disciplinary work, particularly among scientists with little programming experience.

Hypotheses in *ViVA Explorer* can involve any subset of 2500 phenotype variables, and can condition on any of 60 group structures in 18 predefined population subsets (as well as on all experimental protocols). This permits very flexible definition of variance structure models. Results of analyses are ‘web sites’ that can be refined or extended collaboratively at any time.

### Visualization of variance

Our variance structure models *M* :*Y*∼*X*∣*G*:(*S*) are adaptations of the formulas *Y*∼*X*∣*G* supported by linear models in the S and R statistical computing environments [8]**. They are also a mainstay of ***trellis graphics*[21], an influential visualization toolkit in these environments. In trellis graphics, the values (factor levels) of conditioning variables define an array or grid (‘trellis’) of similar visualizations, allowing side-by-side comparison across these values. Thus the conditioning variables yield automatic construction of grids of visualizations.

Side-by-side display is a powerful tool for visualization of variance, although we have not seen it presented or developed in this way. A new emphasis of ViVA is in directly linking visualization with variance structure models, permitting interactive visual exploration of dimensional representations of disorder.

How does ViVA differ from existing visualization systems? For example, the Clinical suite of tools in Spotfire [24][25], a widely-used visualization framework, provides summary statistics, relation plots, and many other types of charts. Most statistical computing environments provide these functions as well. Although it doesn’t provide more functions or analytical power than these systems, ViVA is novel and has some strengths.

**Figure 3:**
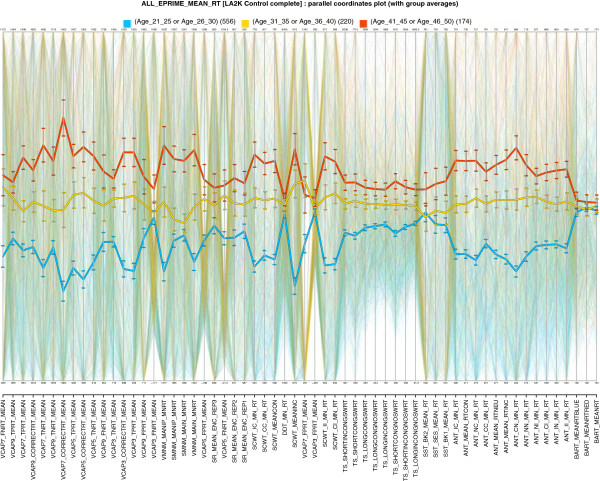
**The ravages of age: every mean reaction time variable in the CNP database exhibits slowing with age.** The three phenotype profiles (thick lines showing averages by age group) are consistently ordered across all variables, exhibiting a progressive increasing of reaction times with age in *every* task. This is an example of *VISOVA*, an integration of parallel coordinates plots [21] with analysis of variance: if horizontal trajectories in the diagram were removed, and made to emphasize the group averages for each variable (column), this would resemble a parallel ANOVA display. However VISOVA also selects a variable ordering so that highly-correlated variables are clustered together.

**Figure 4:**
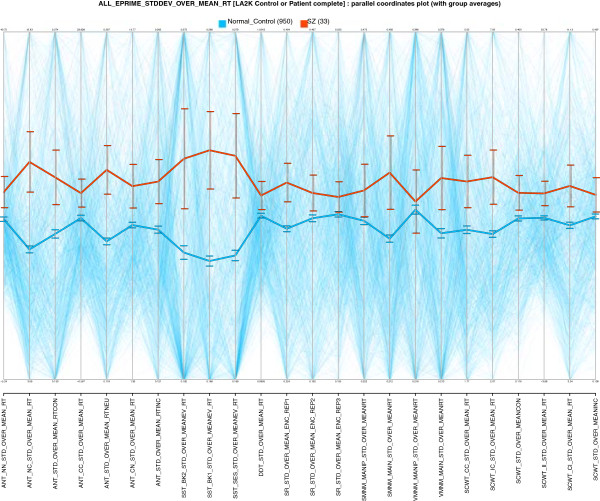
**Phenotype profiles of controls (blue) and Schizophrenia patients (red) on the Coefficient of Variation (CV = *σ */ *μ *) for every Reaction Time measure in the database possessing both *μ * and *σ *.** In other words, for every available RT measure with *μ* and *σ*, Schizophrenia patients have higher RT Variability. Despite the diversity of tasks (SMNM, DDT, ANT, SCWT, SR, SST), the differences are consistent and exceed the standard error (error bars).

ViVA focuses on visualization of variance. It ties variance structure models directly to visualization, permitting exploration of hypotheses across groups; this is unique. For example, Figures 3 and 4 below show examples of *VISOVA* (to be outlined below)—a novel integration of ANOVA, clustering, and parallel coordinates. Also it anticipates some questions and includes additional results in reports with sets of visualizations, rather than individual plots. Out of concern over the likelihood of misinterpretation, however, it supports only hypothesis exploration, not hypothesis testing.

ViVA goes to lengths to make exploration effortless. It consists of about 50,000 lines of Python code, which is equally split between web interface and back-end data management. The latter involves automation of updates in a data extraction and cleaning pipeline, implementation of group structures and subpopulations, creation of useful extra tables and fields, and visualization functions that have been improved over time. These things can be implemented in any environment, but LA2K is complex, and ViVA provides three exploration modes (Atlas, Viewer, Explorer), which led to a custom design.

Existing research practice is very different—it begins with a basic hypothesis and concludes with statistical hypothesis tests. Generally the hypothesis concerns a small set of variables, involving at most a few tables, with questions like those behind the design of LA2K. The first step is specific (and deliberately limited) selection of variables from the data dictionary, followed by data download, manual cleaning and reformatting as spreadsheets, and loading into a statistical computing environment. Visualization is not heavily used if at all, partly out of principle and partly because of the perceived energy expenditure required to generate useful displays. Statistical computing environments provide trustworthy hypothesis testing.

Although ViVA includes no hypothesis testing, the time required to complete the other research steps—from selection of populations and variables to obtaining analysis reports—is at most a few minutes. The resulting visualizations immediately detect mistakes in variable selection and identify concerns about variable distributions and assumptions. Visualization can instantly suggest discarding or refining a hypothesis. ViVA cannot automate human hypothesis development, but it can support it with exploration.

## Results and discussion

We illustrate hypothesis exploration in ViVA—with visualization of variance—with several example scenarios. These applications highlight points that motivated the development of LA2K, such as the value of phenotype profiles that cut across disciplines. However the main thing they illustrate is how exploration can refine hypotheses. Although it cannot validate or prove hypotheses, then, ViVA can ground them in data—and introduce forces of natural selection that yield better hypotheses. This section offers a few examples to illustrate what ViVA has to offer for hypothesis development and visualization of variance. More examples are available at http://www.hypweb.org.

### Sample hypothesis: age and reaction time

Consider a rough hypothesis that reaction time (RT) is affected by age. We can explore this hypothesis by considering *all* Mean RT values (joined across tables). Figure 3 shows a *VISOVA* (parallel coordinates/ANOVA) visualization for a table containing all key *MEAN RT* variables, essentially a sequence of variance structure models *VISOVA* : ∼*ReactionTime*∣*Age*:(*LA*2*Kcontrol*), in which each column shows the effect of age on one (independent) RT variable. This axis labels shows all of the MEAN RT variables included and the columns representing variables are clustered according to correlation similarity. In other words, each column represents the value range of a single variable, and trajectories across the columns give the sequence of RT values for a single subject.

VISOVA displays extend parallel coordinates with group structure. Individual group averages for the variables are also superimposed as thicker lines along with standard error bars; the colors reflect the 7 age ranges used as groups. In other words, the continuous age variable here has been used to obtain discrete population subgroups. The thick red line at the top shows the average for subjects aged 41–50, while the blue line near the bottom shows the average for subjects aged 21–30. Thus a thick line represents a phenotype profile (pattern of average values) for a particular group. The gradual increase of RT over these age groups is consistent across all Mean RT variables, suggesting an ongoing process of decline (progressive increase in mean reaction time) with age, regardless of task or type of RT measure (e.g., congruent vs. incongruent RT). This continuum in this effect suggests that a mathematical model of decline might be possible.

The recent article [26] notes that Age is often incorrectly treated as a ‘nuisance variable’—a quantity of little perceived interest or relevance, yet which must be statistically controlled as it might indirectly affect quantities of interest. It asserts that controlling for age can give distorted views of disease processes; many psychosocial factors are influenced by the stage of life. Nuisance assumptions are difficult to check, but this example shows that doing it can be imperative, and visualization can help.

The results here encourage exploration of still stronger hypotheses. For example, the same variance structure model could be specialized with other factors—such as demographic factors like gender or ethnicity, and behavioral factors like smoking habits. Any of the 60 factors in ViVA could be checked. Changes to the hypothesis above to incorporate new group structure or include other variables only require changing selections in the interaction menu. The interface encourages exploration of assumptions.

### Sample hypothesis: timing and schizophrenia

Hypotheses regarding the characterization of Schizophrenia in terms of interval timing have emerged recently [27][28]. Various deficits in temporal processing are associated with symptoms of the disorder. It is interesting to explore whether LA2K can provide some support for this association.

The database includes a large number of temporal phenotypes, many in the form of reaction time (RT) measurements. These RT variables include mean and standard deviation (a digest of multiple trials), but provide nothing further for interpretation. Consequently, ViVA was constructed to augment these with both SNR (signal-to-noise-ratio =*μ*/*σ*) and CV (coefficient of variation =*σ*/*μ*) variables, normalizations that permit useful visual comparison of values. In other words, ViVA augments the database variables with useful related measures of variance.

ViVA can explore hypotheses about differences in RT variability (RT CV) exhibited in Schizophrenia. An advantage of RT CV instead of RT alone is that it is less sensitive to the underlying reaction time distribution. Some related published work is in [29]**, including arguments for using CV in measuring reaction time **[30]. Although there has been recent interest in response time variability as a measure, there is almost no existing work relating RT variability and Schizophrenia.

Figure 4 shows a VISOVA display of CV values that cut across the database, with the variance structure model *VISOVA* : ∼*ReactionTimeCV*∣*G*:(*LA*2*Kcontrol*+*LA*3*Cpatient*) where *G* is an ad hoc group constructed to include control subjects and Schizophrenia patients. In this plot, Schizophrenia patients have uniformly higher CV values, and the differences exceed the standard error. This cannot validate hypotheses, but it is a demonstration of how one can ground hypotheses in data. It might encourage exploring other measures of variability—such as RT SNR and RT FF (Fano factor =*σ*^**2**^/*μ*).Exploration of variance structure—with ViVA’s automatic augmentation of the database with CV variables—in this case suggests new hypotheses, with interesting results. Despite the broad set of RT measures in Figure 4 (all CV measures in the CNP database—spanning SMNM, DDT, ANT, SCWT, SR, and SST), with few exceptions the differences are greater than the standard error (error bars). Furthermore, more exploration suggested that RT CV is not clearly age-dependent (unlike MEAN RT in Figure 3), encouraging investigation of RT CV as a supplementary measure to RT.

### Sample hypotheses: ethnicity profiles

Next we offer two examples in which ViVA raised doubt about hypotheses. Both involve ethnicity.

**Figure 5:**
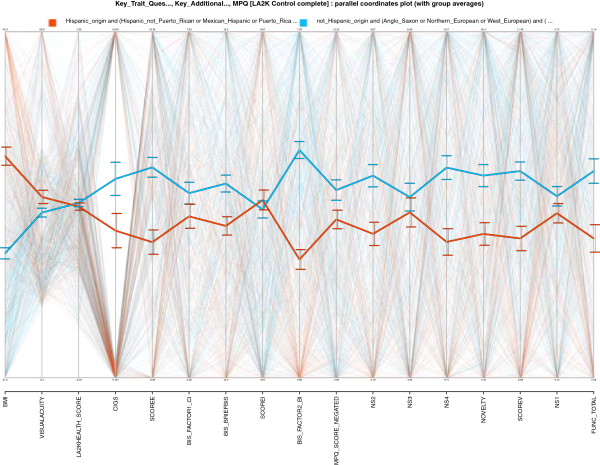
**This VISOVA display shows ‘ethnicity profiles’ across novelty seeking and health indicators for both Hispanic (orange) and non-Hispanic (green) subjects.** For clarity the ethnicity of Hispanic subject’s parents were constrained to be Hispanic, and ethnicity of non-Hispanic subjects were constrained to be European (Anglo-Saxon, Western European, or Northern European). Health did not appear to be influenced by ethnicity: there are no apparent difference on the aggregate LA2K Health Score (a tally of health problems), despite differences in BMI. Also, differences in Novelty Seeking (NS) and impulsivity-related indicators contradicted an initial hypothesis of higher NS scores in Hispanic subjects.

The recent study [31] suggested possible links between health and ethnicity. This conclusion was based on a discovery that a region of the human genome that codes for many antibodies has sections that can be absent, and this variation can depend on ethnicity. Thus, ethnicities might have different health profiles.LA2K was designed in part for analysis of Hispanic ethnicity, and about 40% of its subject population is Hispanic. The CNP database also has some variables related to health, offering a way to ground the health hypothesis in data. Figure 5 shows overall phenotype profiles (patterns of values for each group) across phenotypes related to health and Novelty Seeking/impulsive behavior.

The health phenotypes in Figure 5 shows no differences between Hispanic and non-Hispanic subjects. To put this in perspective, it is not hard to find ethnic differences that could have links to health. Figure 5 shows clear differences for BMI, so variance structure is evident. However, this variance does not extend to the LA2K Health Score—a sum across 21 items asking about diagnoses with, or treatment for, a number of serious medical illnesses. In other words, no pattern was apparent in *Covariance* :*Health*∼*BMI*∣*Ethnicity*:(*LA*2*Kcontrol*), and exploration did not find any health differences linked to ethnicity.

There are other interesting hypotheses regarding ethnicity that are based in genetics. For example, specific variants of the DRD4 gene have been associated with *Novelty Seeking* (NS) behavior, and a well-known hypothesis is that NS was important for human migration out of Africa 50,000 years ago [32]**. A careful recent analysis **[33]** confirmed an association between the DRD4 2R and 7R polymorphisms and migratory distance. The DRD4 7R polymorphism also has been associated with a variety of phenotypes related to NS, including ADHD and smoking phenotypes. However, there is controversy about these associations, and differing results have emerged in populations from different countries [**[34].An initial hypothesis then might be that subjects of Hispanic ethnicity have higher NS scores. Figure 5 shows that grounding this hypothesis in LA2K suggests the opposite: NS-related summary variables from the TCI yield lower score values for Hispanic subjects. Furthermore, smoking measures were lower for Hispanic subjects. This profile appears robust, consistent across multiple indicators.

However, the LA2K ‘ethnicity’ classification is self-reported, and controls were screened for ADHD. For clarity the ethnicity of subjects was constrained to match both parents in Figure 5. The measures related to Novelty Seeking from the TCI inventory were consistently higher for non-Hispanic subjects. We found support only for negative hypotheses *VISOVA* : ∼*NS*∣*Ethnicity*:(*LA*2*Kcontrol*) relating NS to Hispanic ethnicity. Grounding of both ethnicity hypotheses in data raised only doubts.

Development of ethnicity profiles as a robust form of hypothesis checking is an interesting direction for work with complex phenotypes like NS and health. Furthermore, the eventual incorporation of genome-wide genetic variation data in ViVA will allow investigators to stratify groups based on more precise measures of ancestral background, as opposed to relying solely on self-reported ethnicity. Generally, phenotype profiles fit the phenomics/cross-disciplinary outlook mentioned earlier. Multi-population, multi-phenotype views also have found success before in integrative visualization systems [17]**-**[19].

### Hypotheses about neuropsychiatric statistical architecture: gender and disorder prevalence

As mentioned earlier, large databases and systems like ViVA may become important for developing better neuropsychiatric diagnostics. A noteworthy example is in differences in disorder incidence rates by gender reported in [5], which analyzed patterns of comorbidity in the National Epidemiologic Survey on Alcohol and Related Conditions (NESARC, *n*>40,000). The results not only clarify how disorders affect men and women differently, but also offer an overall statistical architecture for this difference.

**Figure 6:**
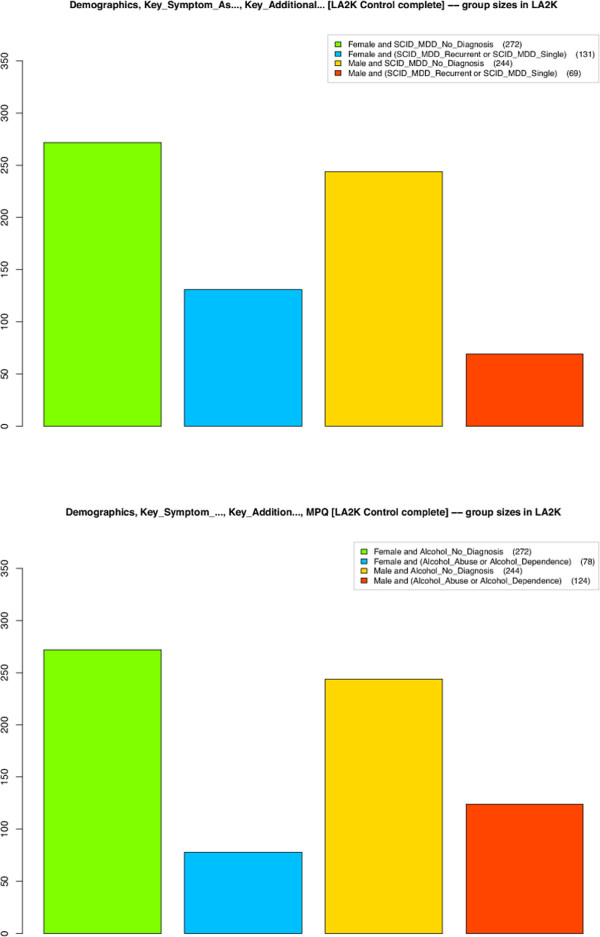
**Internalizing disorders such as MDD (Major Depressive Disorder) are known to have higher prevalence in women, and externalizing disorders such as AAD (Alcohol Abuse and Dependence) to have higher prevalence in men.** ViVA permits rapid verification of this in LA2K controls. Group sizes for MDD are on the left, and AAD on the right (normal females: green, affected females: blue; normal males: orange, affected males: red).

Many studies have found that women have a higher incidence of internalizing (mood and anxiety) disorders, while men have a higher incidence of externalizing (antisocial and substance use) disorders [6]**. This schism is now reflected in the overall internalizing-externalizing dimensional model of DSM-V **[2]. This gender difference is easy to check with ViVA; Figure 6 shows that the prevalence of MDD (Major Depressive Disorder, an internalizing disorder) and AAD (Alcohol Abuse and Dependence, an externalizing disorder) both follow the expected gender-specific liability profile.

Recently a dimensional model of comorbidity among these disorders appeared in [5], with the surprising claim that the comorbidity prevalence of disorders is *identical* in both genders. That is, once the latent gender-specific liability levels are conditioned for, the structure of common disorders is gender-invariant. This result suggests a single overarching disorder structure. It also bypasses a basic problem in analyzing comorbidity—the assumption that the categories of the disorders are valid. If the disorders are dimensional, comorbidity should be dimensional also.

Reported levels of disorder prevalence by gender have varied [6][35][36]**, although the outline of internalizing and externalizing liability has been confirmed. These confirmations were important in the arduous DSM-V development process, which has required years of deliberations and field trials **[2]. The ability to explore the structure of human variation in large databases like NESARC, with systems like ViVA, could be a way to increase consensus and incorporate scientific models into neuropsychiatric practice.

**Figure 7:**
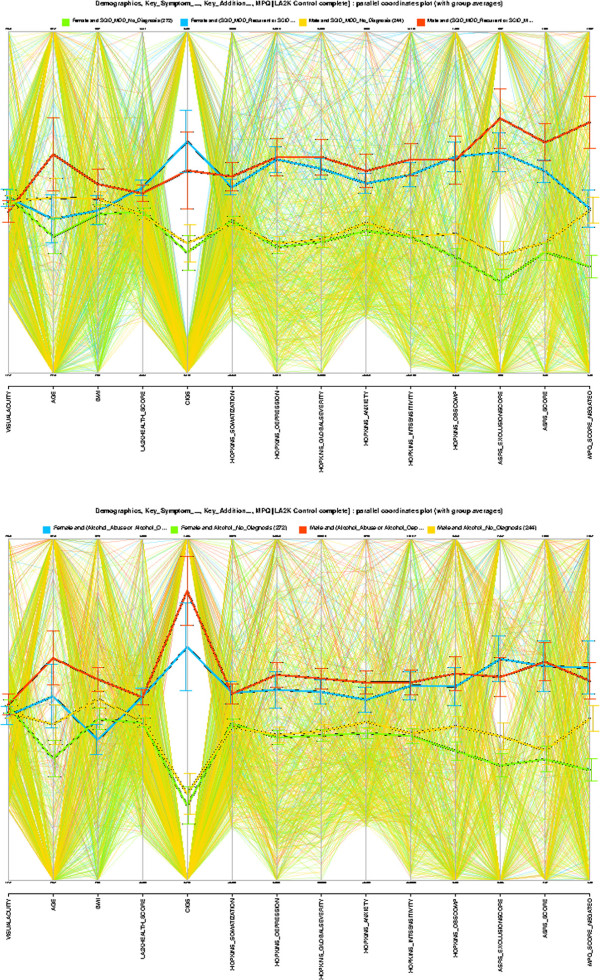
**As a reflection of ‘comorbidity’ of MDD (Major Depressive Disorder) and AAD (Alcohol Abuse and Dependence) with other psychological disorders, we can look at their average profiles against other mental health indicators and measures of psychological stress.** The figures here display the psychological stress profiles of MDD (left) and AAD (right) across summary scores from the Hopkins System Checklist (HSCL-25). They include two summary scores from the Adult ADHD Self-Report Scale (ASRS) in order to track associations with ADHD; and Age and Smoking (Cigs) are also added as checks. The parallel coordinates displays here show similar average profiles. It has been known for some time that internalizing disorders such as MDD (Major Depressive Disorder) have higher prevalence in women than in men, and externalizing disorders such as AAD (Alcohol Dependence) have higher prevalence in men than women (normal females: green, affected females: blue; normal males: orange, affected males: red).

Figure 7 appears consistent with the gender-invariance claimed in [5]. The left image shows the psychological stress/mental health profile for all MDD (Major Depressive Disorder) subjects, and the right shows this for all AAD (Alcohol Abuse and Dependence) subjects, among LA2K controls—where these groups were defined based on a previous diagnosis. The profile is a set of summary scores from the HSCL-25 Hopkins System Checklist, and ASRS ADHD Self-Report Scale, giving a broad assessment of mental health. In both images affected males are shown in red, and females in blue; similarly control males are orange, and control females are green. Age and Smoking are strongly correlated with both MDD and AAD. The average profiles for MDD and AAD are similar across the spectrum of Hopkins and ASRS scores, and the distinctions between male and female subjects are not pronounced. The gender-invariance hypothesis is not contradicted by the data.

## Conclusions

Phenomics and phenotype databases are natural settings for hypothesis exploration. In biomedicine, hypotheses often concern variance structure—patterns of variation in variables when group and population structure are controlled. The abundance of extensions for ANOVA in the statistical and biomedical literature [8] show the importance of variance structure. However ViVA is novel in combining it with visualization as a system, introducing methods like *VISOVA* to integrate visualization and ANOVA.

In ViVA, a variance structure model is an assertion *M* : *Y*∼*X*∣*G*:(*S*) controlling variables *G* in population *S*, representing variance *M* among the variables *Y* and *X*. For example, a model such as *VISOVA* : *Reaction Time*∼*Age*∣*Gender*:(*LA*2*Kcontrol*) represents changes in reaction time by age across healthy male and female populations. There should be significant difference in the association between variables *X* when conditioned on the different groups.

Multi-level models are important in neuropsychiatry for many reasons, including their basic connections with nosology and diagnosis. Among these, a fundamental aspect of conditioning is that it makes hypotheses *differential*—they consider not only a base hypothesis with a single level value, but also alternative level values. For example, a hypothesis could assert that phenotype profiles differ characteristically for each group (i.e., level). Without this differential structure, hypotheses are difficult to falsify, and difficult to make mutually exclusive. As a result they are difficult to verify or contradict, and they all can be ‘right’. This lack of exclusivity, and the difficulty of grounding hypotheses in data, impedes progress [37].

ViVA provides a way, even for scientists with little programming experience, to ‘try hypotheses on for size’ by grounding them in data. It permits selection of any subset of the 2500 database variables, conditioning on any of 60 group structures in 18 predefined populations (as well as on all experimental protocols), using any of a large set of standard variance visualization schemes, without concerns about implementation or details of data cleaning (because these steps are provided by ViVA). To avoid confusion about scientific validity of the results, it is also intentionally limited to hypothesis exploration without hypothesis testing. The exploration process is one of rapid evolution under selection, with stronger hypotheses surviving.

Amidst the deluge of data in which scientists now find themselves, it is vital to integrate relevant information with complex hypotheses. ViVA is an example of ways science can expand from established hypothesis-based processes to more data-driven, discovery-based processes that benefit from the abundance of information.

## Competing interests

The authors declare that they have no competing interests.

## Authors’ contributions

DSP developed the ViVA systems and wrote this article. EC and RMB managed the development of the CNP database, and helped correct and revise the article. All authors read and approved the final manuscript.
